# Study of Determinants of Somatoform Disorders in Children

**DOI:** 10.7759/cureus.36447

**Published:** 2023-03-21

**Authors:** Ganesh Shanker, Indira Sharma

**Affiliations:** 1 Department of Psychiatry, G.S.V.M. (Ganesh Shankar Vidyarthi Memorial) Medical College, Kanpur, IND; 2 Department of Psychiatry, Institute of Medical Sciences, BHU (Banaras Hindu University), Varanasi, IND

**Keywords:** dsm-iv-tr, i.q. of children, psychiatric disorders, scholastic performance, somatoform disorders

## Abstract

Introduction: Somatoform disorders constitute a group of illnesses that present with predominant physical symptoms for which there is no demonstrable etiology, and psychological factors are implicated in initiating, exacerbating, and maintaining the disorder.

Aim and objectives: The aim of this study was to study a host of factors, namely, the I.Q. of children, scholastic performance in the last three years, the past history of medical illness, psychiatric disorders in the family, and life stress in the last year, and to compare these factors with those in matched control subjects. This study looked at the effects of somatoform disorders on children's I.Q., scholastic performance in the previous three years, a history of medical illness, psychiatric disorders in the family, and life stress in the previous year.

Materials and methods: In this case-control study, a sample was selected from the child guidance clinic of the psychiatry outpatient department (OPD) of the University Hospital, Banaras Hindu University, Varanasi, India. The time period of study was from January 2011 to June 2012. All the patients who presented to the child guidance clinic of the psychiatry OPD with one or more somatic symptoms as among their chief complaints were screened. All subjects fulfilling the selection criteria were recruited into the study. An I.Q. test was administered to the children, and the raw score for the verbal and performance tests was calculated. Children above the age of 16 years were assessed with the Wechsler Adult Performance Intelligence Scale.

Results: One hundred fifteen cases that fulfilled the selection criteria were studied. About 14.6% (N = 11) of the patients had a history of significant physical illness as opposed to 12.5% (N = 5) in healthy controls. The scholastic performance in the last three years of the patients group was poorer than that of the control group. The mean number of stressful life events in the patients group was 5.95±1.77 (range: 1-10), and in the control group, it was 1.25±0.43 (range: 1-3).

Conclusion: Compared to controls, patients had a significantly lower I.Q. and poorer scholastic performance in the last three years, a more frequent past history of medical illness, and a greater frequency of life stress in the areas of studies, health, family-related issues, and interpersonal issues.

## Introduction

Somatoform disorders constitute a group of illnesses that present with predominant physical symptoms for which there is no demonstrable etiology, and psychological factors are implicated in initiating, exacerbating, and maintaining the disorder [1]. More recently, other categories of disorders presenting with somatic symptoms have been identified. Diagnostic and Statistical Manual of Mental Disorders, Fourth Edition, Text Revision (DSM-IV-TR) has seven subtypes of somatoform disorders, namely somatization disorder, conversion disorder, pain disorder, hypochondriasis, body dysmorphic disorder, undifferentiated somatoform disorder, and somatoform disorder not otherwise specified. International Classification of Diseases, Tenth Revision (ICD-10) as a reference on the other hand describes seven major categories, namely somatization disorder, undifferentiated somatoform disorder, hypochondriacal disorder, somatoform autonomic dysfunction, persistent pain disorder, other somatoform disorders, and somatoform disorder unspecified. In ICD-10, conversion disorder has been described as a dissociative disorder, in a separate category. The prevalence of somatization disorder in the general population is estimated to be 0.2%-2% in females and 0.2% in males [2]. Conversion disorder is the most common subtype of somatoform disorder.

It is recognized by most authors that the etiology of somatoform disorders is multifactorial and should be understood within a bio-psycho-social-cultural framework [3]. Many etiological factors, which can be broadly categorized into four groups, environmental, psychological, biological, and attitudinal or behavioral, have been implicated in somatoform disorders in children [4]. Although some of the etiological factors, like the temperament of the child, have been studied in the past, there remains a dearth in the research relating to some very important domains of etiological factors, like stressful life events, the I.Q. of patients, and the I.Q. of parents. Freud and Strachey gave the term "conversion" and postulated the conversion of repressed thought into somatic symptoms [5]. Janet (1929) found conversion disorder to be associated with dissociation [6]. It was Briquet who gave us the modern concept of conversion disorder. Somatization is a poorly understood "blind spot" of medicine. Somatoform disorders remain neglected despite functional impairment and economic burden. Conceptual and clinical questions exist about the validity and utility of the concepts. Our aim is to study the determinants of somatoform disorders in children, and it was determined that there is a significant difference between the patient and control groups with respect to a variety of factors, including the children's I.Q., academic performance in the previous three years, past history of medical illness, psychiatric disorders in the family, and life stress in the previous year. 

The timeline of outline changes made during American Psychiatric Association (APA) meetings is as follows: American Psychiatric Association (APA) (1952), DSM-I: The term "conversion reaction" was coined [7]. APA (1968), DSM-II: The term "hysterical neurosis, conversion type" was used. "Briquet syndrome" was also mentioned [8]. APA (1978), DSM-III: For the first time, somatoform disorders were grouped together. The terms "conversion disorder" and "dissociative disorder" were coined [9]. APA (1994), DSM-IV: The terms, conversion and dissociative disorder, were retained, and the criteria for somatization disorder were simplified to make them easier to use in clinical settings [10]. Dell and Campo (2011), DSM-V: The somatoform disorder category is likely to be dropped, but a new category known as "Somatic Symptom Disorder" will be added [11].

The hallmark of management for somatoform disorders is that the primary care physician must establish a long-term relationship with these patients, with regular checkups and a conservative diagnostic and therapeutic stance. Both ongoing psychiatric consultation and group therapy have been found to be effective in reducing the severity of symptoms, and cognitive behavioral therapy may also be effective. Antidepressants should be chosen with few, if any, side effects, and any potentially addictive substances should be avoided.

## Materials and methods

**Figure 1:**
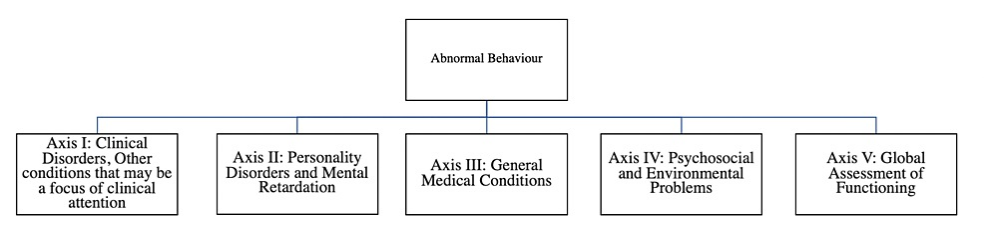
Abnormal Behavior and the Multiaxial Model.

In this case-control study, a sample was selected from the child guidance clinic of the psychiatry outpatient department (OPD) of the Banaras Hindu University Hospital, Varanasi, India. The time period of study was from January 2011 to June 2012. The procedure of purposive sampling was adopted. Children presenting with somatic symptoms as among their chief complaints were examined in detail. Those patients who fulfilled the selection criteria were included in the study. Children with 5-18 years of age, diagnosed with a somatoform disorder as per DSM-TR-IV (American Psychiatric Association, 2000), and consented to the study, were included in the study [12]. Patients with I.Q. <70, the presence of comorbid psychiatric illness, and the presence of major medical illness were excluded from the study. The control group consisted of healthy children who were relatives of patients who attended OPD at the University Hospital. Normal, healthy children with an absence of psychopathology, as indicated by a score of <10 on the childhood psychopathology measurement schedule [13], matched with the patient group for age, sex, education, and socioeconomic status, and who consented to the study, were included in the control group. Patients with an I.Q. <70 and the presence of any psychiatric or major medical illness were excluded from the control group. Sociodemographic data, past and family history, medical illness, and personal history were collected, and a physical and mental status examination was done. Malin's Intelligence scale was used to measure/assess the I.Q. of the patients and control children up to 15 years of age. The test comprises 12 subtests divided into six verbal (i.e., information, comprehension, arithmetic, similarities, vocabulary, and digit span) and six performance (i.e., picture completion, picture arrangements, block design, object assembly, coding, and mazes) tests [14]. A Life Event Scale for Indian Children test was administered for the assessment of life stress in patients. There are 50 life event items on this scale, hierarchically arranged in increasing order of stress score. Each event is assigned a stress score between 0 and 100. The test-retest and inter-rater reliability are adequate. Diagnostic and Statistical Manual of Mental Disorders, Fourth Edition, Text Revision (DSM-IV-TR) (American Psychiatric Association, 2000) [11]: It is a multiaxial classification with five axes as shown in Figure 1.

Kuppuswamy’s Socioeconomic Status Scale accounts for the education, occupation, and income of the family to classify study groups into high, middle, and low socioeconomic strata [15-17]. It is an inherent part of various community-based and many hospital-based studies in India. All the patients who presented to the child guidance clinic of the psychiatry OPD with one or more somatic symptoms as among their chief complaints were screened. All subjects fulfilling the selection criteria were recruited into the study. Details of patients’ sociodemographic data, physical and mental status examinations, reports, investigations, and referrals were recorded in the structured format I. The Malin’s scale was administered to a child (85), and the raw score for the verbal and performance tests was calculated. Raw score was converted into the test quotient (T.Q.). Finally, full-scale I.Q. was obtained by adding the average scores of the verbal and performance tests. This scale was applied when the patient was stable and cooperative, usually after one week of the presentation. Children above the age of 16 years (30) were assessed with the Wechsler Adult Performance Intelligence Scale.

## Results

The mean age of the patient group was 14.44±3.45 years (range: 6-18 years) and that of the control group was 14.17±2.88 years (range: 8-18 years). The mean years of education in the patient group was 7.39±1.98 years (range: 3-14 years) and that of the control group was 8.82±1.31 years (range: 4-14 years). One hundred fifteen subjects who fulfilled the selection criteria were studied. The sociodemographic characteristics of the subjects are depicted in Table 1.

**Table 1:** Sociodemographic Characteristics of Subjects. NS: nonsignificant (p > 0.05), c^2^: Chi-Square, df: degrees of freedom.

Variables	Patient (n = 75)		Control (n = 40)		c^2^	df	p-value
	N	%	N	%			
Sex (Female:Male)	54:21	72.0:28.0	28:12	70.0:30.0	0.051	1	NS
Religion (Hindu:Muslim)	69:6	92.0:8.0	35:5	87.5:12.5	0.611	1	NS
Occupation (Students:Others)	73:2	97.3:2.7	40:0	100:0	1.086	1	NS
Domicile (Rural:Semiurban:Urban)	46:17:12	61.3:22.7:16.0	17:13:10	42.5:32.5:25.0	3.761	2	NS
Socioeconomic Status (Upper middle:Lower middle:Lower)	9:51:15	12.0:68.0:20.0	5:25:10	12.5:62.5:25.0	3.783	2	NS

The mean number of family members in the patient group was 7.58±2.16 (range: 3-17), while in the control group, it was 7.15±1.81 (range: 3-14). The mean number of siblings in the patient group was 3.22±1.42 (range: 1-7), while in the control group, it was 2.95±0.87 (range: 1-8). A little over half of the patients hail from extended families. Almost 1/5th hailed from nuclear families. The remaining 17.3% belonged to joint families (the p-value was significant). The subjects' characteristics of family type and family size are depicted in Table 2.

**Table 2:** Comparison of Family Type and Size in Patient and Control Groups. c^2^: Chi-Square, df: degrees of freedom.

Variables		Patient (n = 75)		Control (n = 40)		c^2^	df	p-value
		N	%	N	%			
Family type	Extended:Joint:Nuclear	39:13:23	51.9:17.3:22.5	14:15:11	35.0:37.5:27.5	6.08	2	0.04
Family size	3	1	1.3	1	2.5	8.21	2	0.01
	4-5	12	15.9	4	10.0			
	6-7	21	27.9	22	55.0			
	> 7	41	54.9	13	32.5			
Total (115)		75		40				

Conversion disorder was the most common (92.1%) subtype of somatoform disorders in patients. More than 80% of patients had an acute onset of illness, and the remaining had an insidious onset of illness. Almost half of the patients had a total duration of illness (TDI) of less than one month. Around one-third had a TDI of one to six months, and the remaining had a TDI of more than six months. The mean duration of illness was 10.82±14.48 weeks (ranging from one day to one year). Episodes of unresponsiveness were the most common presentation, which was found in 52% of patients. The second most common symptom was a headache, in slightly less than half of the patients. Dyspnea and chest pain were the third most common symptoms and were seen in one-fourth of the patients (combined). Other less common symptoms like mutism, loss of vision, downward gaze, and continuous cough constituted less than 20% of the presenting complaints.

The past history of medical and psychiatric illness in patient and control groups is as follows: 14.6% (N = 11) of the patients had a history of significant physical illness as opposed to 12.5% (N = 5) in healthy controls. About 2.6% (N = 2) of patients had jaundice, 2.6% (N = 2) had pneumonia, and 2.6% (N = 2) had typhoid. About 1.3% (N = 1) had acute gastroenteritis; 1.3% (N = 1) had neurocysticercosis; 1.3% (N = 1) had Koch; 1.3% (N = 1) had a urinary tract infection; 1.3% (N = 1) had a seizure disorder; and 1.3% (N = 1) had asthma.

The family history of psychiatric illness in the patient and control groups is as follows: 2.8% (N = 18) of the first-degree relatives (FDRs) of patients had anxiety-related disorders, of which 2.04% (N = 8) had somatoform disorders and 0.76% (N = 3) had generalized anxiety disorders. About 2.07% (N = 3) of patients had FDRs with psychiatric disorders other than anxiety-related disorders, of which 0.76% (N = 3) had major depressive disorders, 0.76% (N = 1) had bipolar affective disorders, and 0.25% had acute psychosis. About 0.41% (N = 1) of the FDRs of controls had anxiety-related disorders. The frequency of anxiety-related disorders was higher in patients with FDRs than in controls. This difference between the patient group and the control group with respect to family history of psychiatric illness in the FDRs of patients and controls was statistically significant, as shown in Table 3.

**Table 3:** Family History of Psychiatric Illness in First-Degree Relatives of Patient and Control Groups. FDR: first-degree relative, c^2^: Chi-Square, df: degrees of freedom.

Variables	Total number of FDR of patients (n = 391)		Total number of FDR of control (n = 240)		c^2^	df	p-value
	N	%	N	%			
Conversion disorder	8	2.0	0	0.0	9.06	2	0.01
Generalized anxiety disorder	3	0.7	1	0.4			
Major depressive disorder	3	0.7	0	0.0			
Bipolar affective disorder	3	0.7	0	0.0			
Acute psychosis	1	0.2	0	0.0			
No illness	373	95.3	239	99.5			
Total	391		240				

The scholastic performance in the last three years of the patient group was poorer than that of the control group. Around 2/3rd of the patients passed the final exams for the last three consecutive years. One-third of the patients were either promoted or failed at least once in the last three years. About 2.6% (N = 2) repeatedly failed. One-tenth (N = 8) failed once in the last three years, and 1/5th (N = 15) were promoted. About 2/5th had an average scholastic performance. About 1/8th had an above-average and good scholastic performance. The frequency of subjects who failed or were just promoted was higher in the patient group than in the control group. This difference between the patient group and the control group with respect to scholastic performance was statistically significant, as shown in Table 4.

**Table 4:** Comparison of Scholastic Performance in the Last Three Years of Patient and Control Groups. c^2^: Chi-Square, df: degrees of freedom.

Variables		Patient (n = 75)		Control (n = 40)		c^2^	df	p-value
		N	%	N	%			
Scholastic performance	Repeated failure	2	2.6	0	0.0	9.29	4	0.05
	Failure	8	10.6	2	5.0			
	Promoted	15	19.9	3	7.5			
	Average	31	41.3	16	40.0			
	Above average	9	12.1	12	30.0			
	Good	10	13.3	7	17.5			

Stressful life events were categorized into six subgroups: health, study, abuse, family-related, interpersonal, and miscellaneous. The majority of the patients had one to two life events in the areas related to health, study, family, and interpersonal relations. Stressful life events (one, two, or three) in the last year related to health, studies, family issues, and interpersonal issues were more frequent in the patient group than in the control group. About 2/3rd of patients had at least one stressful life event related to health in the last one year. One-fourth of patients had two or more health-related events. One health-related event was noted in 1/4th of the controls. At least two stressful life events related to studies were noted in 3/4th of the patient group. About 13.3% of patients had three study-related events, and about 1/4th of patients had one study-related event in the last one year. About 77.5% of control group subjects had no stressful life events related to studies. More than half of the patients had at least one family-related stressful life events. Around 1/3rd of patients had two family-related stressful life events, and 6.5% had three family-related life events. Around 1/3rd of the control group had one family-related life event. Around 3/4th of patient group had one incidence of stressful life events related to interpersonal problems like sibling rivalry and conflict with friends and/or emotionally attached person. Around 10% of patients had two interpersonal life events, and 1.3% had three interpersonal life events. One-fourth of controls had one interpersonal life event. This difference between the patient group and the control group with respect to life events related to health, studies, family, and interpersonal issues was statistically significant. No statistical significance was found between the patient and control groups with respect to abuse and miscellaneous life events, as shown in Table 5.

**Table 5:** Comparison of Various Types of Life Events in Patient and Control Groups. NS: nonsignificant (p > 0.05), c^2^: Chi-Square, df: degrees of freedom.

Variables	No. of events	Patient (n = 75)		Control (n = 40)		c^2^	df	p-value
		No.	%	No.	%			
Health	0	6	8	30	75	56.22	3	0
	1	50	66.7	10	25			
	2	18	24	0	0			
	3	0	0	0	0			
	4	1	1.3	0	0			
Study	0	1	1.3	31	77.5	84.28	3	0
	1	15	20	9	22.5			
	2	49	65.4	0	0			
	3	10	13.3	0	0			
	4	0	0	0	0			
Abuse	0	60	80	38	95	4.97	2	NS
	1	11	14.7	2	5			
	2	4	5.3	0	0			
	3	0	0	0	0			
	4	0	0	0	0			
Family related	0	1	1.3	23	57.5	59.36	3	0
	1	43	57.3	15	32.5			
	2	26	34.9	4	10			
	3	5	6.5	0	0			
	4	0	0	0	0			
Interpersonal	0	13	17.3	30	75	37.82	2	0
	1	54	72	10	25			
	2	7	9.4	0	0			
	3	1	1.3	0	0			
	4	0	0	0	0			
Miscellaneous	0	68	90.7	36	90	0.01	2	NS
	1	7	9.3	4	10			
	2	0	0	0	0			
	3	0	0	0	0			
	4	0	0	0	0			

The mean number of stressful life events in the patient group was 5.95±1.77 (range: 1-10), and in the control group, it was 1.25±0.43 (range: 1-3). The majority (about 85%) of the patients had experienced four to eight life events in the last year. Less than 1/10th of the patients experienced less than four life events, and the remaining patients experienced more than eight life events. Three or more life events were more frequent in the patient group, while up to two life events were more frequent in the patient group. This difference between the patient group and control group with respect to the numbers of life events was statistically significant as shown in Table 6. 

**Table 6:** Comparison of Number of Life Events in Patient and Control Groups. NS: nonsignificant (p > 0.05), c^2^: Chi-Square, df: degrees of freedom.

Variable	Patient (n = 75)		Control (n = 40)		c^2^	df	p-value
	N	%	N	%			
1	0	0.0	29	72.5	104.10	9	0.00
2	2	2.7	10	25.0			
3	4	5.2	1	2.5			
4	10	13.3	0	0.0			
5	17	22.1	0	0.0			
6	11	14.6	0	0.0			
7	14	18.6	0	0.0			
8	13	17.3	0	0.0			
9	3	3.9	0	0.0			
10	1	1.3	0	0.0			

The mean stress score in the patient group was 350.40±100.20 (range: 150-550), while in the control group, it was 74.25±19.66 (40-120). The mean stress score was higher in the patient group than in the control group. This difference between the patient group and the control group with respect to stress score was statistically significant, as shown in Table 7.

**Table 7:** Stress Score in Patient and Control Groups. df: degrees of freedom.

Variable	Patient (n = 75)		Control (n = 40)		t-value	df	p-value
	Mean	SD	Mean	SD			
Stress Score	350.40	100.249	74.25	19.662	17.212	113	0.00

## Discussion

In the present study, it was observed that the I.Q. of patients was lower than that of controls. A sizable proportion of 34.7% (N = 26) had I.Q. in the borderline range. These observations concur with those made by other workers [18]. Few workers had studied I.Q. and scholastic performance in patients with somatoform disorders. In a recent study from Chandigarh [19] on somatoform disorders and dissociative disorders, I.Q. was significantly higher in somatoform disorder patients than in those in the dissociative disorder group. This finding suggests that patients with somatoform disorders may be heterogeneous with respect to intellectual functioning. The authors opined that the findings were in conformity with the earlier concept of hysteria (which is now included in dissociative disorders). The latter was understood as a form of emotional and cognitive immaturity with a lower intellectual repertoire. It was also observed that scholastic performance was lower in the patient group as compared to the control group, which was reflected in failures, both single and repeated, and more patients being promoted despite failing the examination. The immediate environment of the patient is likely to have an impact on the child's mind. Thus, the effect of mental illness of first-degree relatives of the children was examined in the patients.

It is interesting to note that first-degree relatives were observed to have higher morbidity related to anxiety disorders such as conversion disorders and generalized anxiety disorder. The presence of conversion disorder in the family would provide a non-stigmatizing background for the development of a similar illness in the patient. The findings of the present study are in line with the study by Sharma (2005) [17], as in their study, 22.5% (N = 9) of patients had a history of conversion disorder or epilepsy in the family, among neighbors, or friends. Since witnessing a relative with conversion disorder or epilepsy was fairly common, it has to be emphasized again that children learn by identification and imitation, which explains conversion disorder in parents and their children more clearly [20].

Past histories of medical illness may promote the development of negative emotions toward bodily symptoms, which may lead to the development of somatoform disorders in susceptible children. When life stress in the recent past (i.e., in the last year) was examined, it was noted that most categories of life events (health, studies, family-related issues, and interpersonal issues) were found to be more frequent in the patient group than in the control group. The total stress score related to these events was also higher in the patient group than in the control group.

The findings of our study are in line with observations made by Malhotra (1993) [13], who studied life stress in 80 children with psychiatric disorders. Malhotra [13] observed that the sick group encountered more serious life events, as reflected in their having a greater stress score in the year preceding the onset of symptoms. Sick children also experienced greater stress at a younger age (four to five years) and more undesirable events as compared to normal controls.

It was observed that in the patient group, parenting was grossly deficient in the domains of food, clothing, housing, medical care, parent-child communication, support, routine, education, recreation, creative activities, social activities, rules, managing problem behavior, career guidance, and security. In the same line, it was noted that scholastic performance was lower in the patient group as compared to the control group, reflected by more failures, single as well as repeated, and more patients being just promoted. Thus, in view of the above observations, the research hypothesis (H1) is largely rejected, and it is concluded that there is a significant difference between the patient and control groups with respect to a host of factors, namely the I.Q. of children, scholastic performance in the last three years, past history of medical illness, psychiatric disorders in the family, and life stress in the last year. Although meaningful conclusions may be drawn from this study, a significant limitation of our study is the small sample size, and future studies should be carried out using larger patient populations.

## Conclusions

This study examined the effects of somatoform disorders on the I.Q. of children, scholastic performance in the last three years, the past history of medical illness, psychiatric disorders in the family, and life stress in the last year. One hundred fifteen cases that fulfilled the selection criteria were studied, with 14.6% having a history of significant physical illness as opposed to 12.5% in healthy controls. The mean number of stressful life events in the patient group was 5.95±1.77 (range: 1-10), and in the control group, it was 1.25±0.43 (range: 1-3). The results showed that the patient group had a significantly lower I.Q. and poorer scholastic performance than the control group and a greater frequency of life stress in areas of studies, health, family-related issues, and interpersonal issues. More comprehensive assessments need to be studied further to learn more about somatoform disorders in various age groups.
